# Proximal GATA-binding sites are essential for human *HSD3B1* gene transcription in the placenta

**DOI:** 10.1038/s41598-017-04133-6

**Published:** 2017-06-27

**Authors:** Tsai-Chun Lai, Hsiao-Fang Li, Yu-Shian Li, Pei-Yu Hung, Ming-Kwang Shyu, Meng-Chun Hu

**Affiliations:** 1 Graduate Institute of Physiology, National Taiwan University College of Medicine, Taipei, 100 Taiwan; 2 Department of Obstetrics and Gynecology, National Taiwan University Hospital, Taipei, 100 Taiwan

## Abstract

The enzyme 3β-hydroxysteroid dehydrogenase/isomerase (3β-HSD) is involved in the synthesis of active steroid hormones. Two human 3β-HSD isoforms are expressed in a tissue-specific pattern. *HSD3B1* (type I) expression is essential to produce progesterone for pregnancy maintenance. To understand the mechanisms of human *HSD3B1* activation in the placenta, 2.2 kb of 5′-flanking sequence and 5′-deletions were fused to the luciferase reporter gene and transfected into human JEG-3 cells. The proximal −238/+337 sequence had the highest promoter activity. Two GATA elements were identified at −106/−99 and −52/−45. Mutations of either sites greatly reduced promoter activity in JEG-3 cells, demonstrating the importance of GATA sites. EMSA revealed the specific binding of GATA2 and GATA3 to the GATA sequences at −106/−99 and −52/−45. ChIP assays demonstrated the association of GATA2 but not GATA3 with the GATA-binding regions of the *HSD3B1* promoter in JEG-3 cells. GATA2 knockdown significantly reduced *HSD3B1* expression in JEG-3 cells; however, GATA3 knockdown increased *HSD3B1* expression. Western blot analysis revealed high levels of GATA2 but not GATA3 in human placental tissues. This study identified GATA motifs as essential control elements for *HSD3B1* transcription and GATA2 as a novel transcriptional regulator of *HSD3B1* expression in the human placenta.

## Introduction

The enzyme 3β-hydroxysteroid dehydrogenase/isomerase (3β-HSD) regulates an essential step in the biosynthesis of steroid hormones, including mineralocorticoids, glucocorticoids, and sex steroids^1^. Multiple isoforms of 3β-HSD have been identified in humans and rodents. These isoforms are encoded by distinct genes and are expressed in a tissue-specific pattern^2^. Humans possess two 3β-HSD isoforms, type I (*HSD3B1*) and type II (*HSD3B2*), which share 93.5% identity in their amino acid sequences^3^. *HSD3B2* is exclusively expressed in the classic steroidogenic tissues including the adrenal gland, ovary, and testis, whereas *HSD3B1* is expressed in the placenta and peripheral tissues, including the mammary gland, prostate, and skin^3–5^.

Progesterone is essential for the establishment and maintenance of pregnancy in mammals. The biosynthesis of progesterone from cholesterol is catalysed by two steroidogenic enzymes: cytochrome P450 side-chain cleavage enzyme (P450scc; encoded by *CYP11A1*) and 3β-HSD. During the early stages of human pregnancy, the corpus luteum of the ovary is active in secreting progesterone for the implantation and maintenance of pregnancy^6^. After about 8–10 weeks, large amounts of progesterone are produced by the placenta to sustain the pregnancy. The expression of *HSD3B1* in the placenta is involved in the biosynthesis of progesterone; thus, it is needed for successful pregnancy. Little is known about the molecular mechanisms that regulate *HSD3B1* expression in the placenta. Previous studies have demonstrated that stimulation with cAMP and PMA (phorbol 12-myristate 13-acetate) can increase *HSD3B1* mRNA in human choriocarcinoma JEG-3 cells^7^; however, no response elements have been identified in the promoter of the *HSD3B1* gene. An initial study investigating the regulatory sequences of the *HSD3B1* promoter has demonstrated that some cis-acting elements located in the first intron regulate the basal expression of the *HSD3B1* gene^8^. Furthermore, a distal cis-regulatory element located at −2570/−2518 mediates the specific expression of the *HSD3B1* gene in the placenta^9^. This element contains a specific binding site for transcription enhancer factor (TEF)-5, which is highly expressed in the human placenta, and an overlapping GATA-binding site for a GATA-like protein.

In this study, we searched for novel regulatory elements within the 2.2 kb proximal promoter of *HSD3B1*. We identified two GATA elements located at −106/−99 and −52/−45 that are essential for the functional activity of the *HSD3B1* promoter in JEG-3 cells. Our data revealed that GATA2 and GATA3 are predominant proteins bound to the GATA elements and may play distinct functional roles in the expression of *HSD3B1* in the placenta.

## Results

### Promoter activity of human *HSD3B1* gene in JEG-3 cells

**Figure 1:**
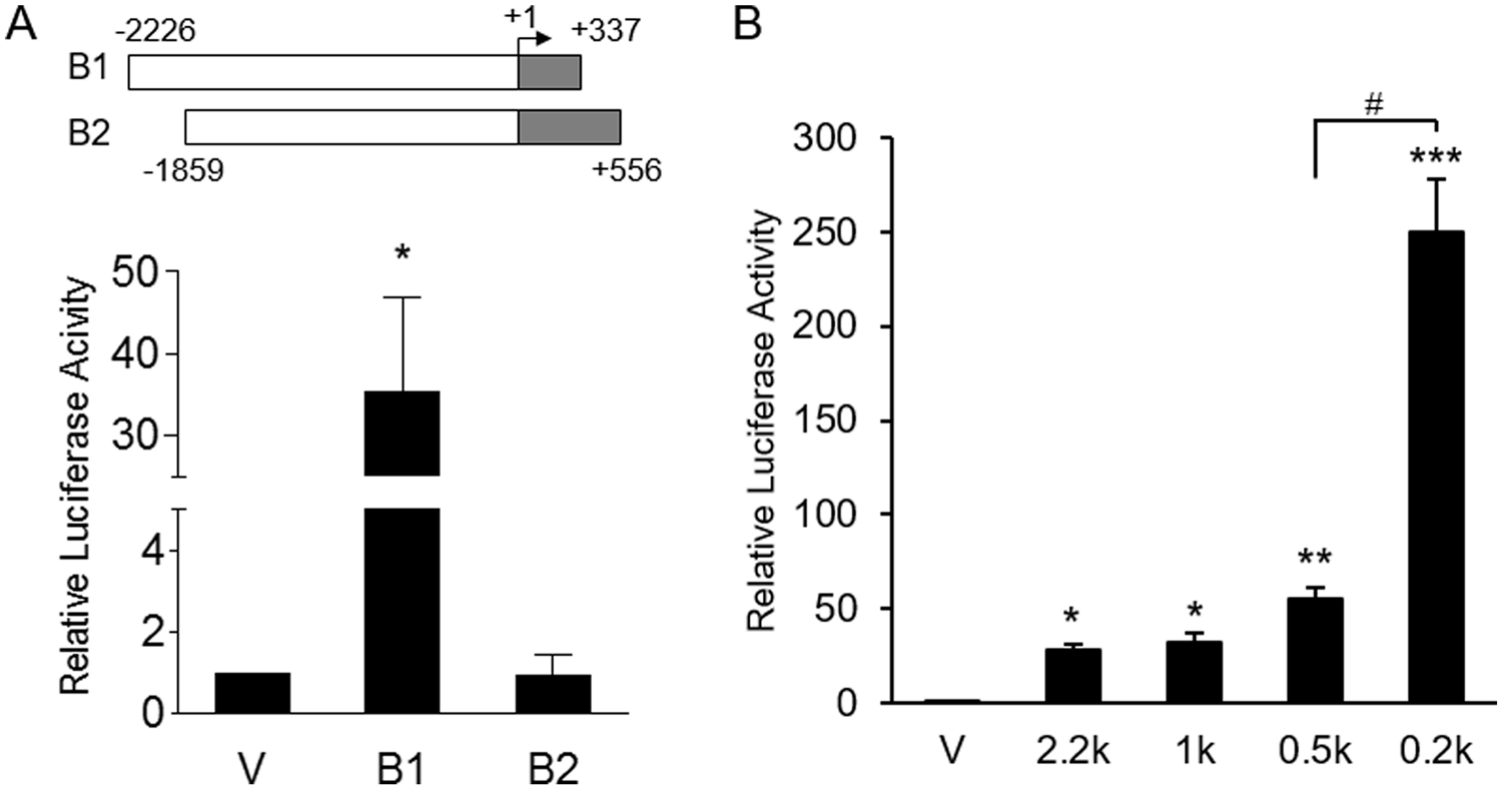
Transcriptional activity of the human *HSD3B1* promoter. (**A**) The human *HSD3B1* (B1: −2226/+337) and *HSD3B2* (B2: −1859/+556) promoter regions (shown on the top) were fused to luciferase reporter to generate promoter-luciferase constructs. “+1” is the transcription start site indicated by the arrow. The *HSD3B1*, *HSD3B2* or empty (v) promoter-luciferase construct was transiently transfected into JEG-3 cells. (**B**) A series of 5′-deleted *HSD3B1* promoter-luciferase constructs were transiently transfected into JEG-3 cells. Cells were harvested after 24 h of transfection and assayed for luciferase activity. Results are presented as the activity relative to the empty vector. Data are means ± SD of five (**A**) or three (**B**) independent experiments. *P < 0.05, **P < 0.01, ***P < 0.001 compared with vector control. ^#^ *P* < 0.05.

To identify novel regulatory elements involved in the placental expression of human *HSD3B1* gene, the 5′-flanking sequence from −2226 to +337 was cloned upstream of the luciferase reporter gene. In addition, the luciferase reporter construct containing the promoter fragment (−1859/+556) from human *HSD3B2* gene was also generated. In human JEG-3 trophoblast cells, the *HSD3B1* −2226/+337 fragment demonstrated a marked increase in transcriptional activity (35 fold) over the basic vector, whereas the −1859/+556 fragment of the *HSD3B2* promoter did not exhibit luciferase activity (Fig. 1A), indicating the specific expression of *HSD3B1* in the placenta. This finding suggests that sequences between −2226 to +337 contain functional elements for the regulation of *HSD3B1* expression in JEG-3 cells. To further localise the regulatory cis-elements in this region, the −2226/+337 fragment was used to generate a series of deletions. As shown in Fig. 1B, the −238/+337 construct had the highest promoter activity in JEG-3 cells. Furthermore, the deletion of 262 bp from −500 to −238 resulted in a drastic increase in transcriptional activity by 4.5 fold, suggesting the presence of negative regulatory elements in this region. These data demonstrate that the −238/+337 basal promoter region contains functional cis-elements responsible for *HSD3B1* expression in the placenta.

### Enhancement of *HSD3B1* promoter activity by GATA2 and GATA3

**Figure 2:**
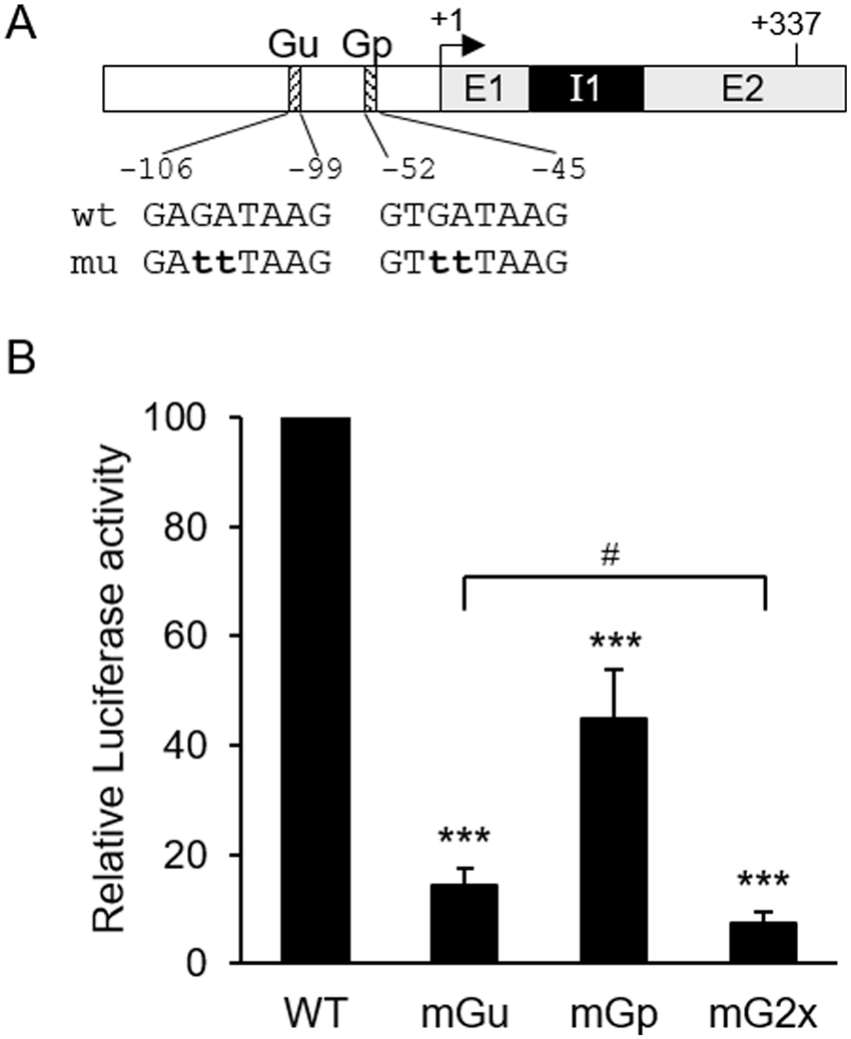
Enhancement of the transcriptional activity of the *HSD3B1* promoter by two putative GATA sites. (**A**) Schematic of the promoter region in the *HSD3B1* gene. “+1” is the transcription start site indicated by the arrow. Two putative GATA-binding sites are indicated as Gu and Gp. Their sequences are shown with mutated nucleotides in lowercase letters. E1, exon 1; I1, intron 1; E2, exon 2. (**B**) JEG-3 cells were transiently transfected with the −238/+337 *HSD3B1* promoter-luciferase construct containing wild-type or mutated GATA sites (either or both sites). Cells were harvested after 24 h of transfection and assayed for luciferase activity. Results are presented as the activity relative to the wild-type construct. Data are means ± SD of five independent experiments. ***P < 0.001 compared with wild-type construct. ^#^ *P* < 0.05.

A database search of the −238/+337 promoter region identified two putative GATA-binding sites at −106/−99 and −52/−45, which are referred to as Gu and Gp, respectively. To determine the role of these GATA-binding sites in the regulation of *HSD3B1* promoter activity, mutated GATA-binding sequences were introduced into the −238/+337 promoter construct (Fig. 2A). As shown in Fig. 2B, mutations of Gu and Gp significantly reduced promoter activity by 87% and 58%, respectively, in JEG-3 cells. In addition, the simultaneous mutation of both GATA sites resulted in a 93% reduction in promoter activity that was significantly lower than the Gu mutation (*P* < 0.05). These results suggested that both the Gu and Gp sites are involved in the regulation of *HSD3B1* promoter activity.

**Figure 3:**
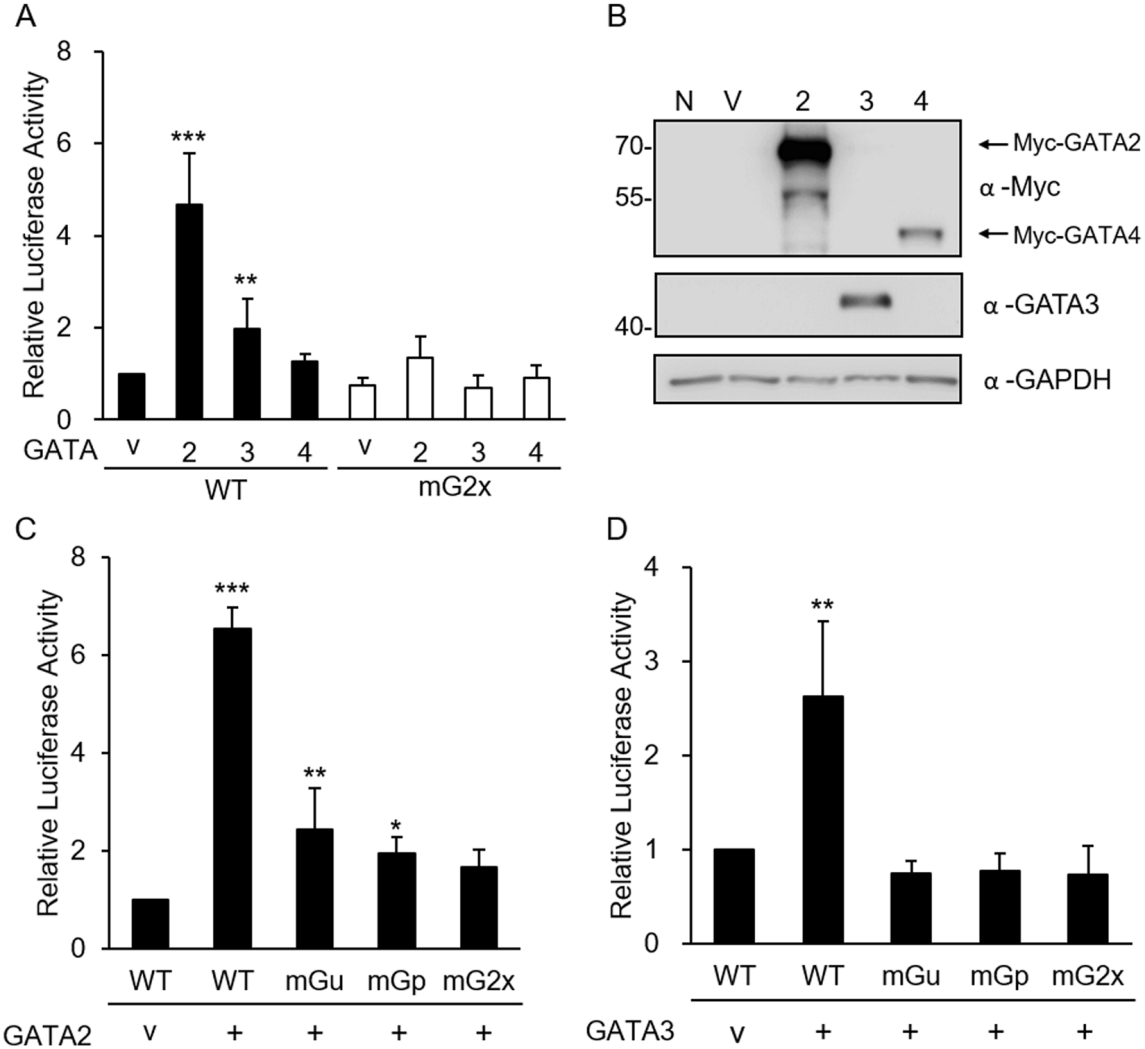
Effects of GATA factors on the transcriptional activity of the *HSD3B1* promoter. (**A**) *HSD3B1* promoter-luciferase constructs containing wild-type or mutated GATA sites were co-transfected into HeLa cells with the expression vector for GATA2, GATA3, GATA4 or empty vector control (v) as indicated in (**A**,**C**,**D**), respectively. Cells were harvested after 24 h of transfection and assayed for luciferase activity. Results are presented as the activity relative to the empty vector. Data are means ± SD of nine (**A**), four (**C**) or five (**D**) independent experiments. *P < 0.05, **P < 0.01, ***P < 0.001 compared with the vector control. (**B**) Expressions of GATA proteins were detected by western blot analysis with anti-GATA3 and anti-Myc for GATA2 and GATA4. N, non-transfected cells; V, vector control. Uncropped images of blots were shown in Supplementary Fig. S3.

The GATA transcription factor family consists of six members, GATA1–GATA6. GATA2 and GATA3 have previously been reported to regulate the expression of a number of genes in trophoblast cells and placental tissues^10^. To determine whether GATA proteins affect *HSD3B1* promoter activity, the −238/Luc construct was co-transfected with GATA expression plasmids in HeLa cells. As shown in Fig. 3A, co-transfection of the −238/Luc construct with GATA2 or GATA3 significantly increased promoter activity, whereas co-transfection with GATA4 did not affect reporter activity. Mutation of the Gu or Gp GATA-binding site resulted in the loss of GATA2- and GATA3-stimulated promoter activity (Fig. 3C and D).

### Expression of 3β-HSD, GATA 2, and GATA3 in the placenta

**Figure 4:**
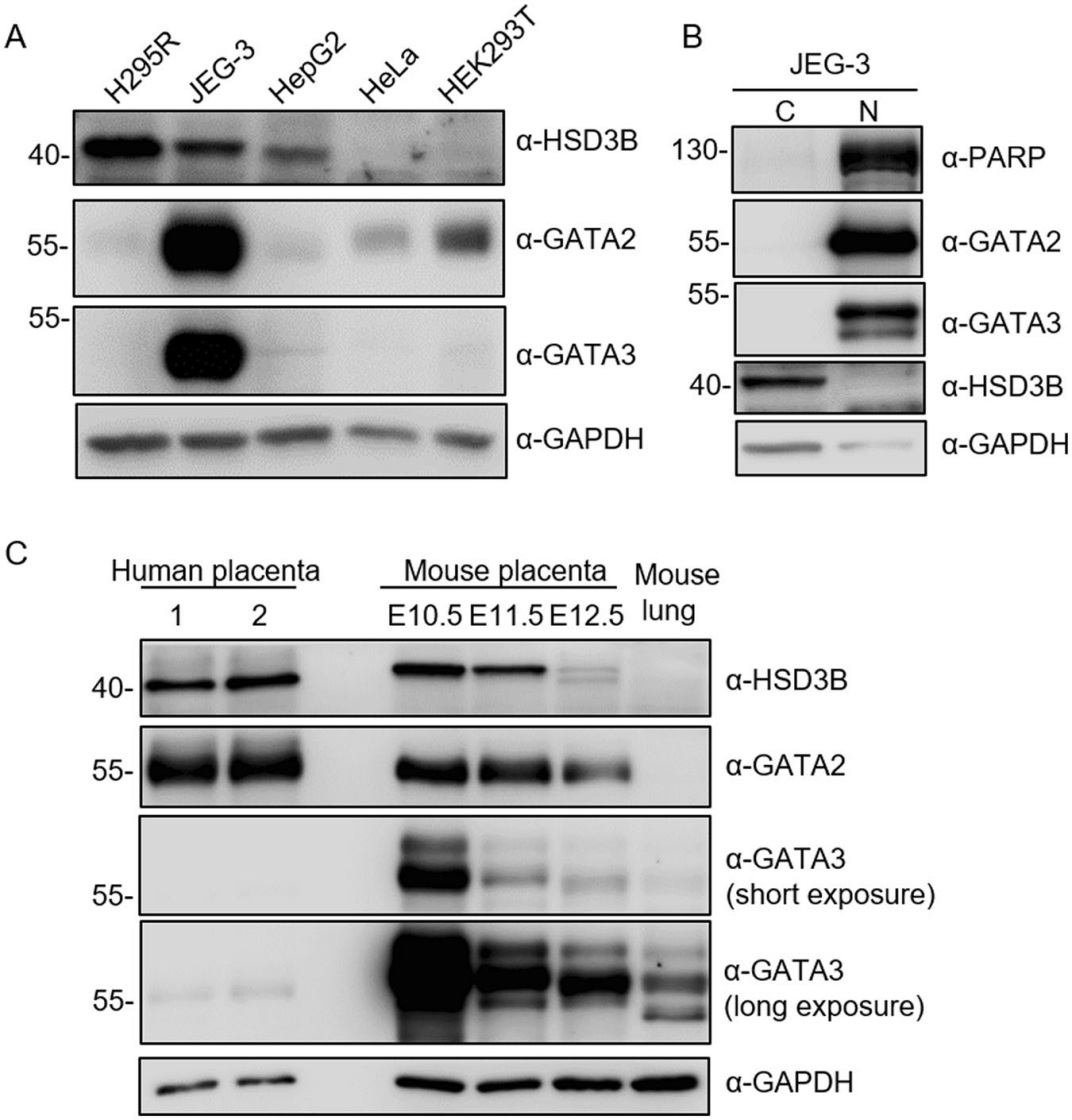
Expression of 3β-HSD, GATA2, and GATA3 in cell lines and tissues. (**A**) Cell lysates from the indicated cell lines were subjected to western blot analysis. (**B**) Nuclear (N) and cytoplasmic (**C**) fractions from JEG-3 cells were examined by western blot analysis. The markers used were PARP for the nucleus and GAPDH for the cytoplasm. (**C**) Protein extracts from two human placental tissues, mouse placenta of E10.5–E12.5, and mouse lung were analysed by western blot analysis. GAPDH was used as a loading control. Uncropped images of blots were shown in Supplementary Fig. S4.

As shown in Fig. 4A, we confirmed the expression of 3β-HSD protein in JEG-3 cells by western blot analysis. The 3β-HSD type 2 is the isoform expressed in human adrenals^3^; thus, a high amount of 3β-HSD protein was detected in H295R human adrenocortical carcinoma cells (Fig. 4A). In addition, Fig. 4A shows that GATA2 and GATA3 proteins were present at high levels in JEG-3 cells but at low to undetectable levels in H295R, HepG2, HeLa, or HEK293T cells. This is consistent with earlier studies showing the high expression of GATA2 and GATA3 in JEG-3 cells but not in H295R cells^11, 12^. Cellular fractionation experiments demonstrated that GATA2 and GATA3 proteins were exclusively present in the nuclear fraction of JEG-3 cells (Fig. 4B).

The expressions of 3β-HSD, GATA 2, and GATA3 were examined in placental tissues by western blot analysis. Mouse 3β-HSD 6 is orthologous to human 3β-HSD 1, which is the only isoform expressed in the placenta and skin^13^. Consistent with previous studies, we found that 3β-HSD protein was highly expressed in mouse placenta on E10.5 and gradually decreased from E11.5–E12.5 (Fig. 4C). Similar to 3β-HSD, protein levels of GATA2 and GATA3 were high in mouse placenta on E10.5 and declined thereafter. However, GATA3 protein level was markedly decreased on E11.5. The protein expressions of 3β-HSD and GATA2 were also high in human placental tissues; conversely, GATA3 protein was detected at a very low level (Fig. 4C).

### Analysis of GATA2 and GATA3 binding to the GATA elements

**Figure 5:**
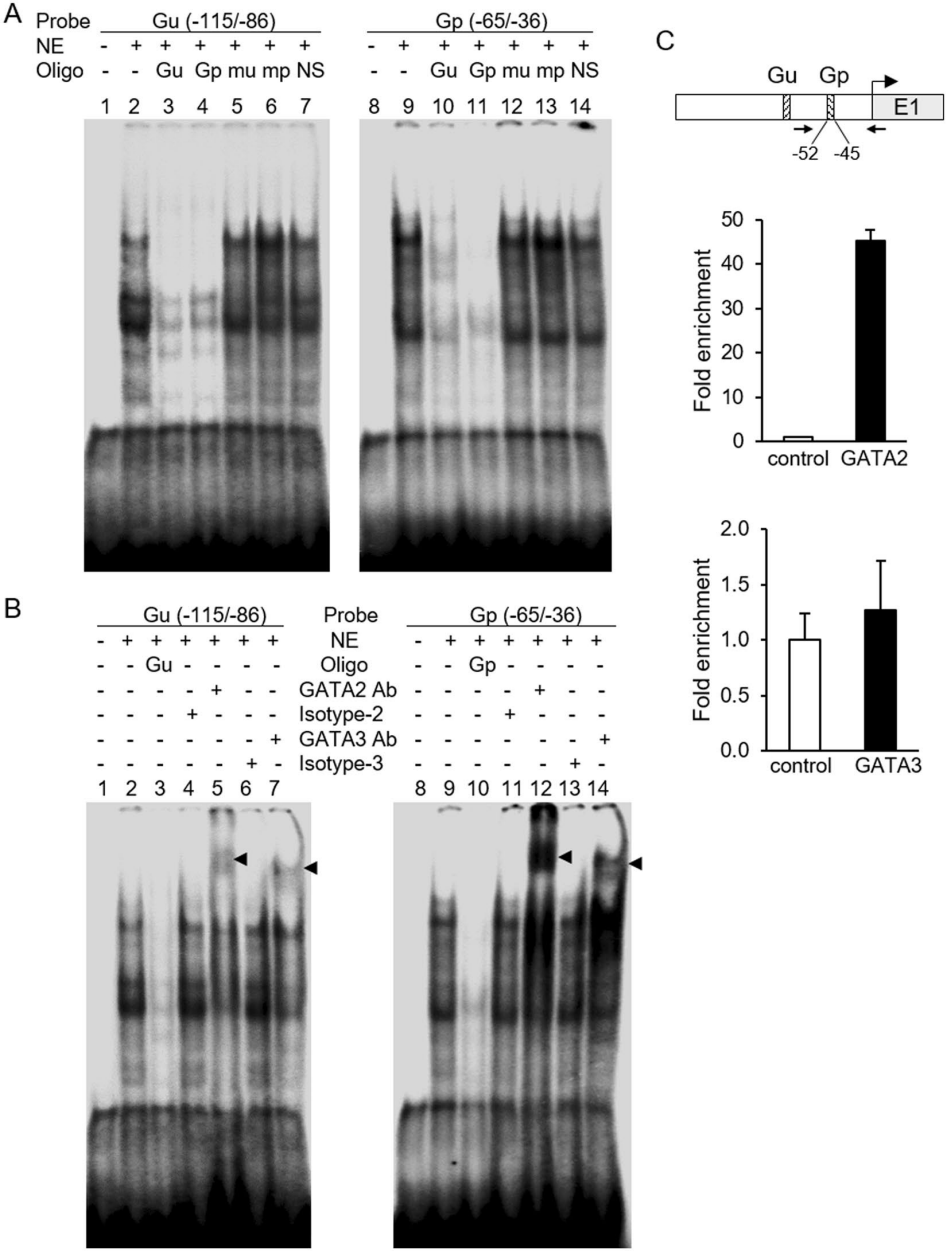
Analysis of GATA2 and GATA3 binding to sites at −106/−45 in the *HSD3B1* promoter. EMSA was performed with JEG-3 nuclear extract (NE) using two radiolabelled probes containing the putative GATA-binding site Gu (−115/−86) or Gp (−65/−36). (**A**) Competition experiments were performed by incubation with a 100-fold excess of the unlabelled oligonucleotides Gu, Gp, mutated Gu (mu), mutated Gp (mp), or non-specific sequence (NS). (**B**) The anti-GATA2 or anti-GATA3 antibody was added to the binding reaction, and the respective isotype antibody was used as a control. Supershift bands are indicated by the arrowhead. (**C**) JEG-3 cells were subjected to ChIP assay with the indicated antibodies or control IgG and analysed by qPCR using a primer set (indicated by arrows) covering the Gp site. The PCR-amplified fragment is 107 bp (−96~+11). Representative results of two independent experiments are shown. Data are means ± SD of triplicate qPCR analyses. Uncropped images of blots were shown in Supplementary Fig. S5.

We performed electrophoretic mobility shift assay (EMSA) to examine the potential nuclear proteins involved in binding to the GATA-binding sites. Synthetic oligonucleotides containing the GATA site Gu (−115/−86) and Gp (−65/−36) were used as probes. Nuclear extracts from JEG-3 cells formed DNA-protein complexes with Gu probe (Fig. 5A, lane 2) that were competed by a 100-fold excess of unlabelled Gu (lane 3) or Gp (lane 4) probe. Similarly, protein complexes formed from JEG-3 nuclear extracts and Gp probe were competed by an excess of unlabelled Gu (lane 10) or Gp (lane 11) probe. Probes with mutation of the GATA-binding sequences (mu and mp) shown in Fig. 2 did not compete with the complexes (lane 5, 6, 12, and 13), indicating that the GATA sequence is required for protein binding. With Gu or Gp as the probe, a supershift was observed by the antiserum to GATA2 or GATA3, but not by the control serum (Fig. 5B). This suggests the involvement of GATA2 and GATA3 in the DNA-protein complex observed in EMSA. Furthermore, we used ChIP assays to confirm the presence of GATA2 or GATA3 at the endogenous promoter in JEG-3. As shown in Fig. 5C, GATA2 occupancy was observed in the GATA-binding region of the *HDS3B1* promoter, whereas GATA3 was not found to be associated with this region.

### Effects of GATA2 or GATA3 knockdown on *HSD3B1* expression

**Figure 6:**
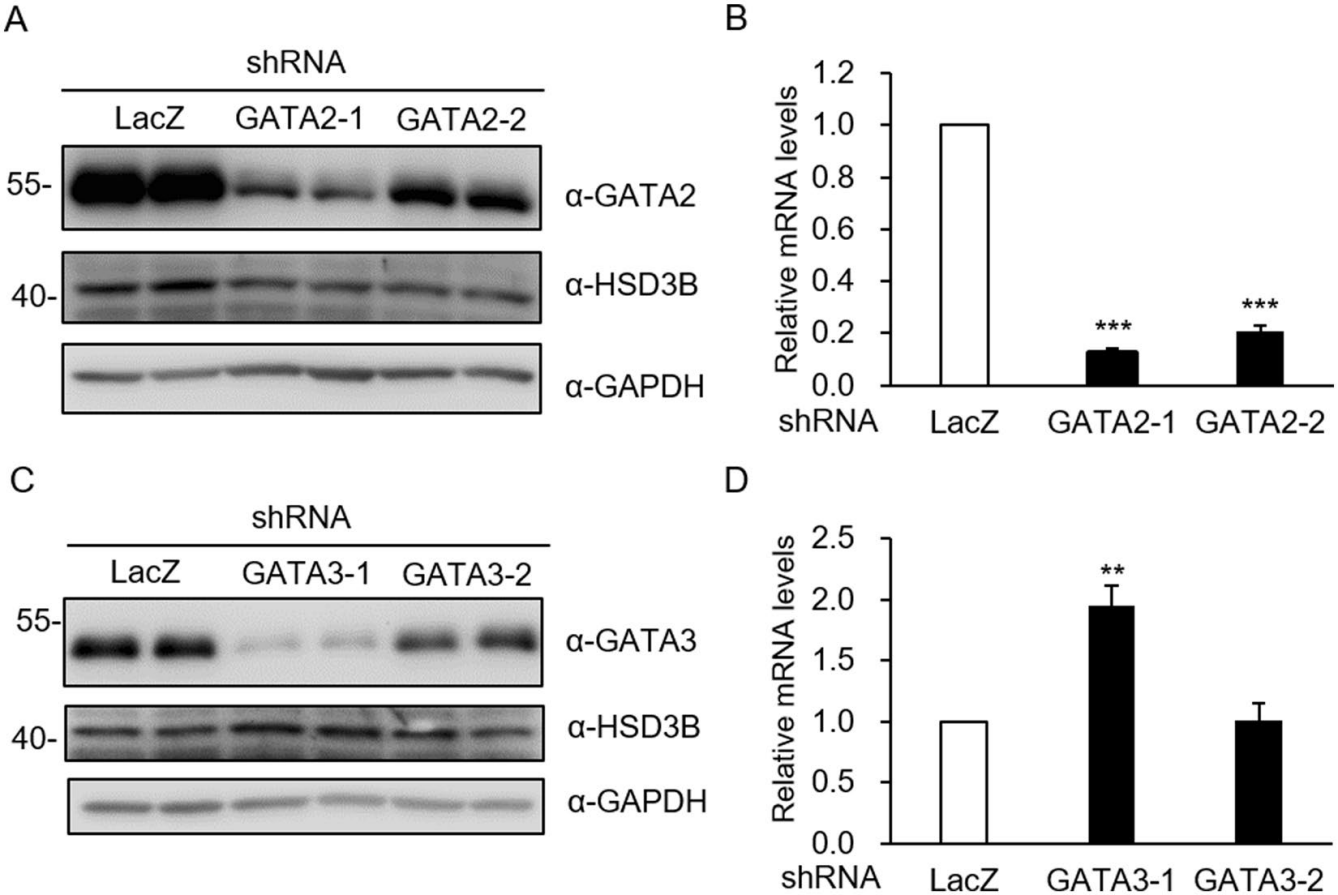
Effects of GATA2 or GATA3 knockdown on *HSD3B1* expression. JEG-3 cells were transfected with shRNA specific for GATA2, GATA3, or LacZ (negative control). (**A**,**C**) Cell lysates were analysed by western blot analysis. (**B**,**D**) RNA was extracted and quantified by qRT-PCR. Values are means ± SEM of three independent experiments. **P < 0.01, ***P < 0.001 compared with the control LacZ (Student’s t-test). Uncropped images of blots were shown in Supplementary Fig. S6.

To determine the roles of GATA2 and GATA3 in regulating *HSD3B1* expression, we used shRNA to knock down GATA2 or GATA3 in JEG-3 cells. As shown in Fig. 6A and B, knockdown of GATA2 markedly reduced *HSD3B1* mRNA levels by ~80% and protein levels by ~70% in JEG-3 cells. In contrast, we observed that GATA3 knockdown resulted in a 2-fold increase in the mRNA and protein levels of *HSD3B1* (Fig. 6C and D). The results suggest that GATA2 and GATA3 may play distinct functional roles in *HSD3B1* transcription.

## Discussion

Human *HSD3B1* is involved in the production of placental progesterone for maintenance of pregnancy. Although several cis-acting elements have been implicated in the transcription of *HSD3B1*, the transcriptional mechanisms that regulate its basal expression in the placenta remain unknown. Here, we found that the proximal −238/+337 sequence of *HSD3B1* exhibited high promoter activity in human choriocarcinoma JEG-3 cells. In this region, two GATA elements were identified at −106/−99 (Gu) and −52/−45 (Gp). Mutation of the GATA-binding sites at Gu or Gp greatly reduced promoter activity in JEG-3 cells. Our findings in this study strongly suggest that the proximal GATA motifs are important for trophoblast-specific expression of the *HSD3B1* gene, and GATA2 and GATA3 play a role in the regulation of *HSD3B1*.

GATA transcription factors have conserved zinc finger domains that bind to the consensus DNA motif [(A/T)GATA(A/G)] to regulate the transcription of downstream genes. In mammals, six GATA factors are expressed in a tissue-specific manner and involved in the regulation of cell-specific gene expression. Earlier studies have indicated that GATA2 and GATA3 are the isoforms predominantly expressed in placental trophoblast cells, regulating the expression of a number of genes such as placental lactogen I, proliferin, and syncytin^10, 14, 15^. Our study showed that both GATA2 and GATA3 proteins were abundantly expressed in JEG-3 trophoblast cells and placental tissues (Fig. 4). GATA2 and GATA3 exhibited similar temporal expression patterns to HSD3B in mouse placenta. Furthermore, overexpression of GATA2 or GATA3 significantly stimulated *HSD3B1* promoter activity in non-trophoblast cells (HeLa cells), whereas GATA4 had no effect on *HSD3B1* promoter activity (Fig. 3). Mutations of GATA-binding sites significantly impaired GATA2- or GATA3-induced *HSD3B1* promoter activity. In addition, EMSA experiments demonstrated the specific binding of GATA2 and GATA3 proteins to the GATA sequences at −106/−99 and −52/−45. These data suggest that GATA2 and GATA3 can regulate the transcription of *HSD3B1* through the GATA-binding sequences.

In EMSA, we observed that the two GATA probes (Gu and Gp) seemed to bind with a stronger affinity to GATA2 than to GATA3 (Fig. 5B). ChIP-qPCR assays further verified the binding of GATA2 at the GATA-binding regions of the *HSD3B1* promoter in JEG-3 cells. GATA2 knockdown in JEG-3 cells resulted in a sharp reduction in the expression of *HSD3B*. Together, these findings reveal that GATA2 is the predominant protein recruited to the GATA motifs at −106/−45 to stimulate *HSD3B1* gene expression in JEG-3 cells. In addition to the placenta, the expression of *HSD3B1* has been identified in peripheral tissues including the mammary gland, prostate, and several human cancer cell lines^3–5^. The LNCaP human prostate cancer cells have been shown to express low levels of endogenous *HSD3B1* ^5, 16^. GATA2 ChIP-seq data derived from LNCaP cells are available in the GEO (Gene Expression Omnibus) database under accession number GSE69043^17^. Analysis of the ChIP-seq datasets for LNCaP cells revealed GATA2 ChIP enrichment in the regulatory regions of the *HSD3B1* locus between −267 and +104 (Supplementary Fig. S1), covering the two GATA-binding sites identified in this study. This is consistent with our ChIP-qPCR results of GATA2 in JEG-3, strongly indicating that GATA2 may be necessary for the basal transcription of the *HSD3B1* gene.

In contrast to GATA2, the levels of *HSD3B1* mRNA and protein were increased by GATA3 knockdown in JEG-3 cells, indicating that GATA3 may negatively regulate the expression of *HSD3B1*. EMSA experiments showed that both GATA2 and GATA3 were able to bind to the GATA elements at −106/−45 *in vitro* (Fig. 5B), suggesting that GATA2 and GATA3 may occupy identical GATA-binding regions of the *HSD3B1* locus but exert distinct activities in *HSD3B1* expression. Similar findings demonstrating that GATA factors exhibit different functions through shared chromatin sites were also observed in the transcriptional regulation of the *Gata2* gene. During mouse erythroid differentiation, GATA1 and GATA2 directly regulate *Gata2* transcription. GATA1 represses *Gata2* transcription by displacement of GATA2 from certain GATA-binding sites in the upstream region (−77, −3.9, −2.8, and −1.8 kb) and an intron region (+9.5 kb) of the *Gata2* locus^18–20^. GATA2 is transcriptionally active in these regions, indicating that it positively autoregulates transcription. The process of “GATA switch” was also found in the development of the placenta. GATA3 was reported to repress the *Gata2* gene by occupying the −3.9 kb and +9.5 kb regions of the *Gata2* locus in undifferentiated trophoblast cells^21^. The displacement of GATA3 by GATA2 at these regions induces *Gata2* transcription during trophoblast differentiation^21^. If a similar GATA switch occurs in the *HSD3B1* locus, the concentration of GATA2 and GATA3 may be an important determinant in *HSD3B1* gene regulation. In the present study, we showed that GATA3 protein was scarce in human placental tissues (Fig. 4C). In contrast, GATA2 protein was present at high levels, suggesting the binding of GATA2 to the GATA sites in the promoter to induce *HSD3B1* transcription.

Although both GATA3 and GATA2 were abundant in JEG-3 cells, GATA3 binding to the GATA sites at −106/−45 was not observed in ChIP assays, indicating that this region may not be involved in GATA3-mediated *HSD3B1* repression. Piao *et al*.^22^ identified a GATA3-binding motif in a silencer element that represses the transcriptional activity of the *HSD17B1* promoter in choriocarcinoma cells. By using a transcription factor binding site search program, we identified another putative GATA element present between −558 and −547 of the *HSD3B1* promoter (Supplementary Fig. S2). Mutation of the GATA sequence in this region increased the promoter activity in JEG-3 cells, indicating that this motif is a potential silencer of the *HSD3B1* gene. However, the specific association of GATA proteins to this GATA element was not detectable by EMSA analysis. Whether other GATA3-binding sites are present in the *HSD3B1* locus and participate in the regulation of *HSD3B1* transcription remain to be determined.

The expressions of *CYP11A1* and *HSD3B1* are required for the synthesis of progesterone in the human placenta. A previous study identified a GATA element at −475/−470 that is required for the activation of the *Cyp11a1* promoter in mouse trophoblast giant cells^23^. The study demonstrated via EMSA that GATA2 is the predominant protein bound to this GATA site^23^. In this study, we found that two GATA-binding motifs located at −106/−45 are essential for the activation of the *HSD3B1* promoter in human trophoblast cells. Our data demonstrate that GATA2 binds to the GATA motifs and acts as the critical transcriptional activator of the *HSD3B1* gene in trophoblast cells. In addition, GATA3 seems to exert suppressive effects on *HSD3B1* gene transcription. Together, GATA2 and GATA3 may play a critical role in the regulation of placental progesterone production.

## Methods

### Plasmids

The 5′-flanking regions of human *HSD3B1* (−2,226/+337) and *HSD3B2* (−1,859/+556) were cloned into the pGL3-Basic luciferase vector to generate pHSD3B1-2.2k-Luc and pHSD3B2-1.8k-Luc, respectively. pHSD3B1-1k-Luc was constructed by inserting the *Mlu*I-*Swa*I fragment (−1069/+337) into pGL3-Basic. pHSD3B1-0.5k-Luc was constructed by cloning the PCR-amplified −500/+337 fragment from pHSD3B1-2.2k-Luc into pGL3-Basic. pHSD3B1-0.2k-Luc was constructed by inserting the *Mlu*I-*Puv*II fragment (−238/+337) into pGL3-Basic. Mutations of the GATA-binding sites of pHSD3B1-0.2k-Luc or pHSD3B1-1k-Luc were generated by PCR-based site-directed mutagenesis. Human cDNAs of GATA2 and GATA4 were PCR-amplified and ligated to expression vector to generate Myc-tagged GATA2 and Myc-tagged GATA4, respectively. The expression plasmid pcDNA3-GATA3 for human GATA3 was a generous gift from S.-C. Miaw (Graduate Institute of Immunology, National Taiwan University College of Medicine).

### Human tissues and animals

Human placental tissues were collected at term from the Department of Obstetrics and Gynecology, National Taiwan University Hospital (NTUH). The study protocol was approved by the institutional review board (IRB: 201406055RINC) at NTUH. The informed consent was obtained from all subjects. Animal experiments were approved by the National Taiwan University College of Medicine and College of Public Health Institutional Animal Care and Use Committee. All methods were performed in accordance with the relevant guidelines and regulations. C57BL/6 J mice were mated and checked for vaginal plugs the next morning. Noon on the day the vaginal plug was observed was considered embryonic day 0.5 (E0.5). Pregnant female mice were killed by CO_2_ at the indicated times in the figure. Uterine horns were removed, and the placenta was separated from the embryo.

### Cell culture and transfection

JEG-3 cells were maintained in DMEM/F12 supplemented with 10% foetal bovine serum. Authenticity of JEG-3 cell line was confirmed using short tandem repeat (STR) profiling by Promega (Madison, WI, USA). The test was performed by using the PromegaGenePrint® 10 System and analysed by ABI PRISM 3730 GENETIC ANALYZER and GeneMapper® Software V3.7. HeLa cells were grown in DMEM containing 10% foetal bovine serum. Cell transfections were performed by GenJet™ *In Vitro* DNA Transfection Reagent (Ver. II) (SignaGen).

### Luciferase assay

Cells were subcultured 24 h before transfection in 24-well plates at a density of 10^5^ cells/well. Cells were transfected with 100 ng of reporter plasmid and 10 ng of the control reporter phRLuc. After 24 h, cells were harvested, and luciferase activity was determined using the Dual-Glo Luciferase Assay System (Promega). The results were normalised to internal *Renilla* luciferase activity. Data were obtained from at least three independent experiments. The significance of differences between group means was determined using one-way ANOVA followed by Newman-Keuls post-test. The statistical analysis were performed with GraphPad Prism 6 software (GraphPad Software).

### Cellular extraction, subcellular fractionation, and western blot analysis

Total cell lysates were prepared by lysing cells in modified RIPA buffer [25 mM Tris/HCl pH 7.6, 150 mM NaCl, 1% NP-40, 1% sodium deoxycholate, 0.1% SDS, 5 mM EDTA, 1 mM EGTA, 5 mM DTT (dithiothreitol), 2 mM PMSF, and 10 μg/ml leupeptin]. Cytoplasmic and nuclear fractionations were performed. Initially, cells were lysed in buffer A (10 mM HEPES pH 7.9, 10 mM KCl, 1.5 mM MgCl_2_, 0.5% NP-40, 5 mM DTT, 2 mM PMSF, and 10 μg/ml leupeptin) on ice for 30 min. After centrifugation (13,000 *g* for 10 min at 4 °C), the supernatant was collected as the cytoplasmic fraction, and the nuclear pellet was washed with buffer A and centrifuged. The pellet was then resuspended in buffer B (20 mM HEPES pH 7.9, 0.42 M NaCl, 0.2 mM EDTA, 1.5 mM MgCl_2_, 25% glycerol, 5 mM DTT, 2 mM PMSF, and 10 μg/ml leupeptin). After sonication and centrifugation (13,000 *g* for 10 min at 4 °C), the supernatant was retained as the nuclear fraction. Proteins were separated by SDS-PAGE and subjected to western blot analysis. Antibodies against GATA3 and HSD3B were purchased from Santa Cruz Biotechnology, and GATA2 was from Abcam. Antibodies against Myc and GAPDH were purchased from Millipore, and PARP was from BD Pharmingen. The signal was detected by WesternBright ECL HRP substrate (Advansta).

### Electrophoretic mobility shift assay (EMSA)

Oligonucleotides containing the GATA-binding site from the human *HSD3B1* promoter were end-labelled with [γ-^32^P]ATP and T4 polynucleotide kinase (NEB), annealed, and purified with illustra™ MicroSpin™ G-25 Columns (GE Healthcare). The sense strand sequences of the probes used were Gu: 5′-TATTTTTCTGAGATAAGGATCCCATAGGAG-3′ and Gp: 5′-GCCAGAGATCAAAGTGATAAGGGTTGGGCC-3′. Nuclear extract (2.5 μg) prepared from JEG-3 cells were equilibrated in binding buffer [10 mM HEPES-KOH pH 7.5, 1 mM EDTA, 50 mM KCl, 1 mM DTT, 0.1% BSA, and 0.5 μg poly(dI-dC)] at 4 °C for 20 min. For competition experiments, a 100-fold molar excess of cold oligonucleotides was added to the mixture. For supershift assays, 2 μg of anti-GATA2, anti-GATA3, or the respective isotype antibody was added to the mixture. Subsequently, the ^32^P-labelled probe was added and incubated at room temperature for 15 min. The binding products were resolved on 6% non-denaturing PAGE (Tris/borate/EDTA) and imaged using a Typhoon FLA 9000 PhosphorImager (GE Healthcare Life Sciences).

### shRNA knockdown

The shRNA-expressing lentiviral plasmids (pLKO.1-shRNA) were obtained from the National RNAi Core Facility (Academia Sinica, Taipei, Taiwan). GATA2 was efficiently targeted with the construct TRCN0000355775 or TRCN0000355776, and GATA3 was targeted with the construct TRCN0000273942 or TRCN0000273991. The shRNA targeting LacZ (TRCN0000072223) was used as a control. Lentiviral particles were prepared as described previously^24^.

### RNA extraction and quantitative reverse transcription (qRT-PCR)

Total RNA was extracted using TRIzol reagent (Thermo Fisher Scientific) and then reverse transcribed into cDNA by First Strand cDNA Synthesis Kit (Thermo Fisher Scientific). qRT-PCR analyses were performed using SYBR® Green PCR Master Mix (Thermo Fisher Scientific) in an ABI StepOnePlus^TM^ machine (Thermo Fisher Scientific). Primer sequences used were as follows: HSD3B1 (forward primer 5′-CGGCTAACGGGTGGAATCTG-3′; reverse primer 5′-CCCCATAGATATACATGGGTCGTAAG-3′) and β-actin (forward primer 5′-GGGAAATCGTGCGTGAC-3′; reverse primer 5′-CAAGAAGGAAGGCTGGAA-3′). The relative quantification of mRNA levels was determined by the 2^−ΔΔCt^ method and normalised to β-actin in accordance with the manufacturer’s instructions (Thermo Fisher Scientific). Triplicate qPCR amplifications were performed for each sample. Data were obtained from three independent experiments. The significance of differences between means of two groups was determined using Student’s t-test.

### Chromatin immunoprecipitation (ChIP)

JEG-3 cells were incubated with 1% formaldehyde in growth medium for 10 min at room temperature, followed by adding glycine to a final concentration 125 mM for 5 min to stop the crosslinking reaction. Cells were then washed and scraped in ice-cold PBS and centrifuged, and the cell pellet was resuspended in ChIP lysis buffer (50 mM HEPES-KOH pH 7.5, 140 mM NaCl, 1 mM EDTA, 1% Triton X-100, 0.1% sodium deoxycholate, 0.1% SDS, 5 mM DTT, 2 mM PMSF, and 10 µg/ml leupeptin) on ice for 10 min. The cell lysate was sonicated with an ultrasonic processor (Sonics & Materials), diluted 1:3 with dilution buffer (50 mM HEPES-KOH pH 7.5, 150 mM NaCl, 1 mM EDTA, 1% Triton X-100, 2 mM PMSF, and 10 µg/ml leupeptin), and incubated with anti-GATA2 or anti-GATA3 antibody. Rabbit or mouse IgG isotype was used as a negative control. After incubation of the immunoprecipitate with Protein G Mag Sepharose Xtra (GE Healthcare) for 4 h, the beads were washed with high salt buffer (20 mM Tris pH 8.1, 2 mM EDTA, 0.1% SDS, 1% Triton X-100, and 500 mM NaCl) and eluted with elution buffer (1% SDS in TE buffer). RNase was added, and the crosslinks were reversed by incubation at 65 °C for 4 h. After proteinase K treatment at 55 °C for 1 h, the DNA was purified using QIAquick Gel Extraction Kit (Qiagen). The purified DNAs were subjected to qPCR using the forward primer 5′-TCCCATAGGAGGAGAGAGCA-3′ and reverse primer 5′-CCTCATTTCCTGTGGCAAGT-3′.

## Electronic supplementary material

Supplementary information
