# Supplementary material for: Proximal GATA-binding sites are essential for human HSD3B1 gene transcription in the placenta
Source: Sci Rep. 2017 Jun 27;7:4271. doi: 10.1038/s41598-017-04133-6 (PMC5487343; doi:10.1038/s41598-017-04133-6)
Supplement: Supplementary file 1 — Supplementary information [file 41598_2017_4133_MOESM1_ESM.pdf]

Supplementary Information

Proximal GATA-binding sites are essential for human *HSD3B1* gene transcription in the placenta

Tsai-Chun Lai, Hsiao-Fang Li, Yu-Shian Li, Pei-Yu Hung, Ming-Kwang Shyu, and

Meng-Chun Hu

GATA2 ChIP-seq: LNCaP cells

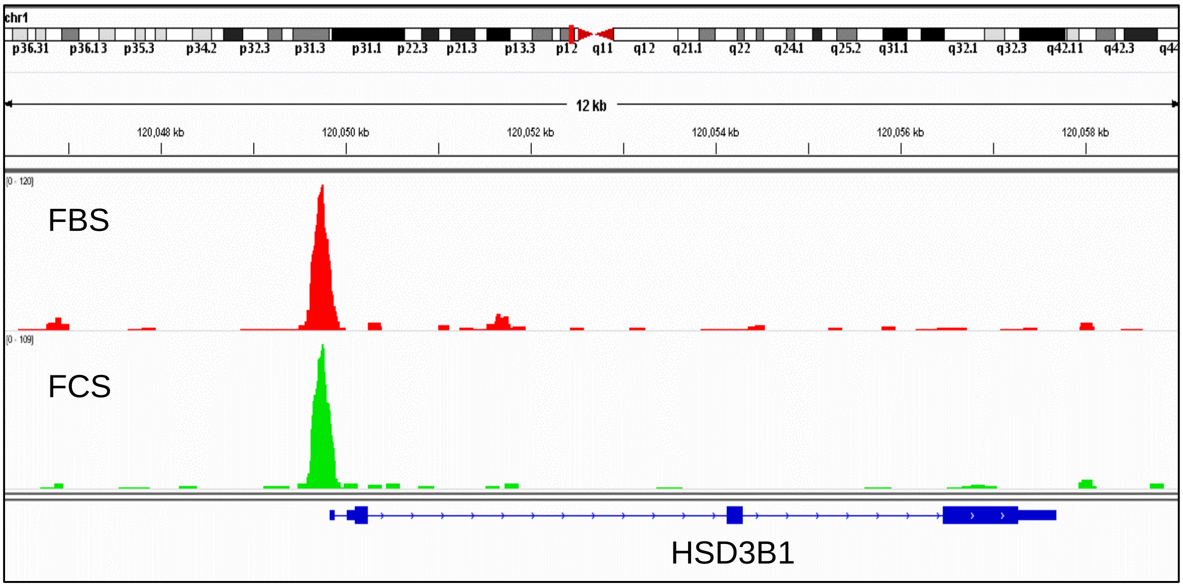

Supplementary Figure S1. Integrative Genomics Viewer shows GATA2 ChIP-seq results using the data set from GEO.

The GATA2 ChIP-seq data of LNCaP cells was published by Zhao et al. (16) and obtained from NCBI GEO database under accession number GSE69043. The GATA2 ChIP-peak was observed around the region -267/+104 of *HSD3B1* gene in LNCaP cells. FBS, foetal bovine serum (in the presence of androgen); FCS, foetal calf serum (in the absence of androgen).

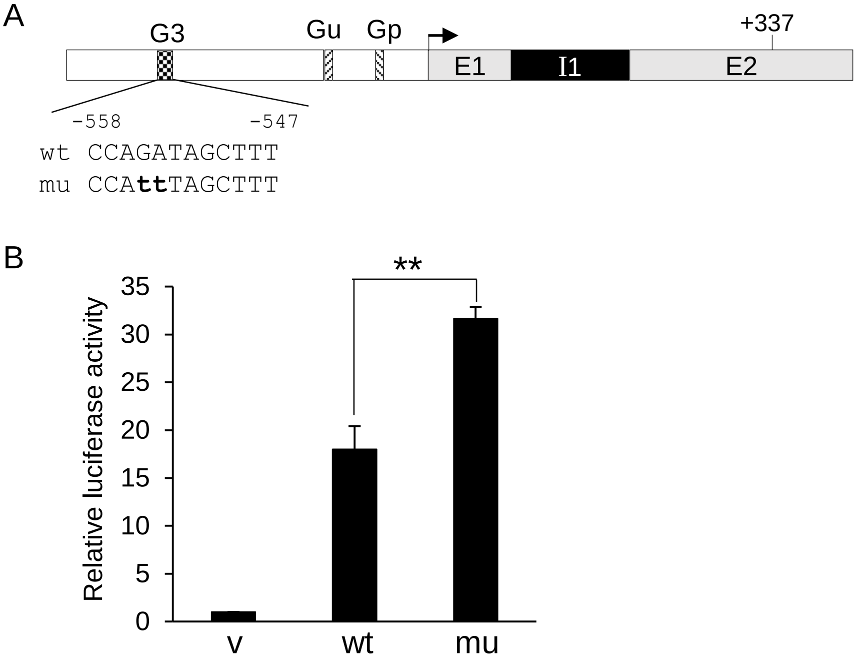

Supplementary Figure S2. Analysis of GATA-like binding site between -558 and -547 in *HSD3B1* promoter.

(A) Schematic of the promoter region in the HSD3B1 gene. “+1” is the transcription start site indicated by the arrow. Two putative GATA binding sites, Gu and Gp, are shown in Fig. 2. The sequences of a GATA-like binding site at -558/-547 (G3) are shown with mutated nucleotides in lowercase letters. (B) JEG-3 cells were transiently transfected with the -1069/+337 HSD3B1 promoter-luciferase construct containing wild-type or mutated GATA site in the G3. Results are presented as the activity relative to empty vector. Data are means ± SD of three independent experiments.). ***P* < 0.01 .

B

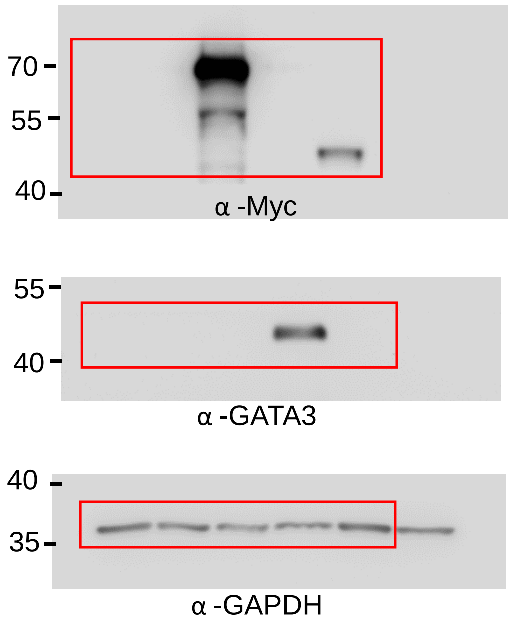

Supplementary Figure S3. Uncropped images of blots presented in the main Figure 3.

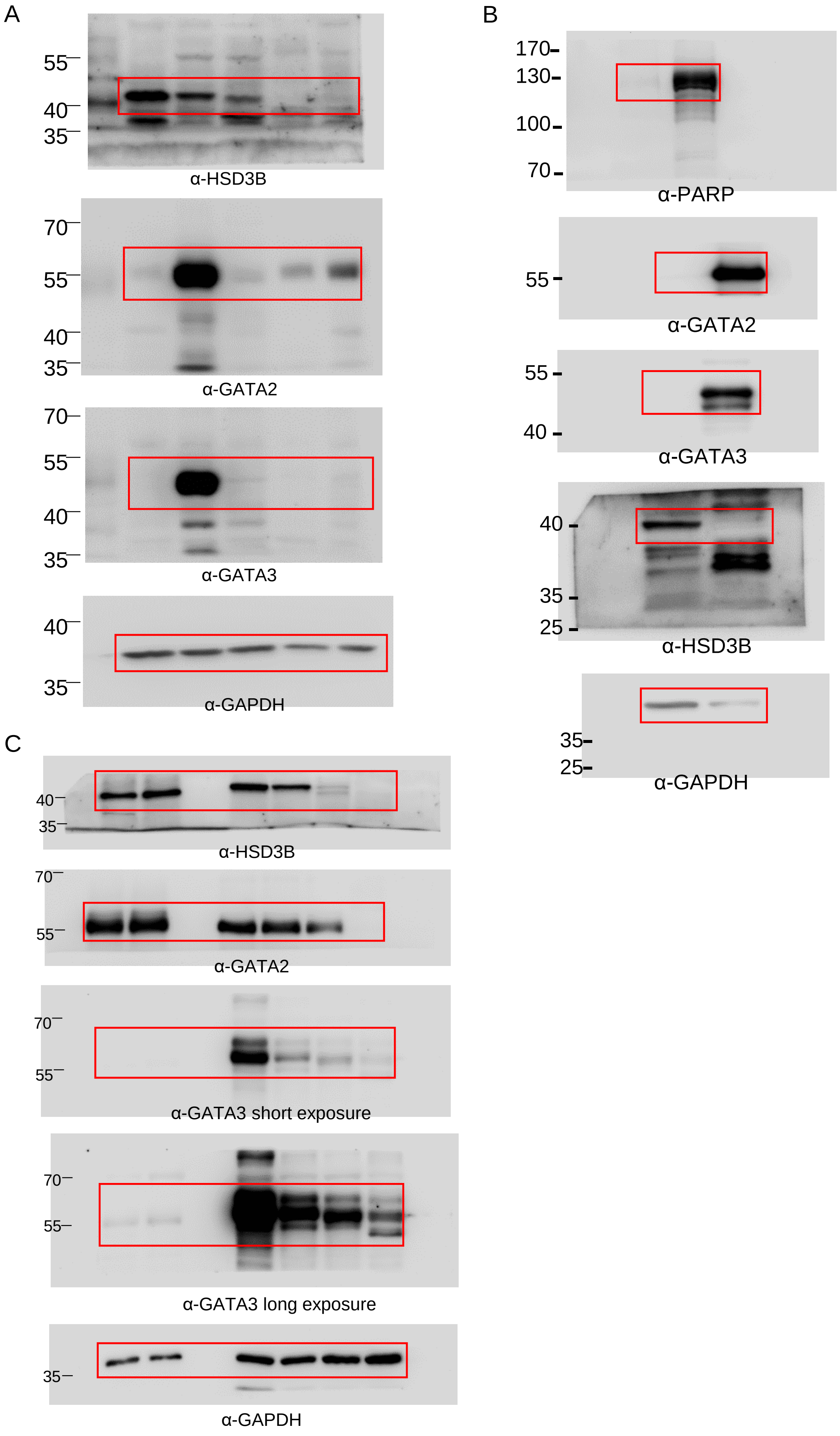

Supplementary Figure S4. Uncropped images of blots presented in the main Figure 4.

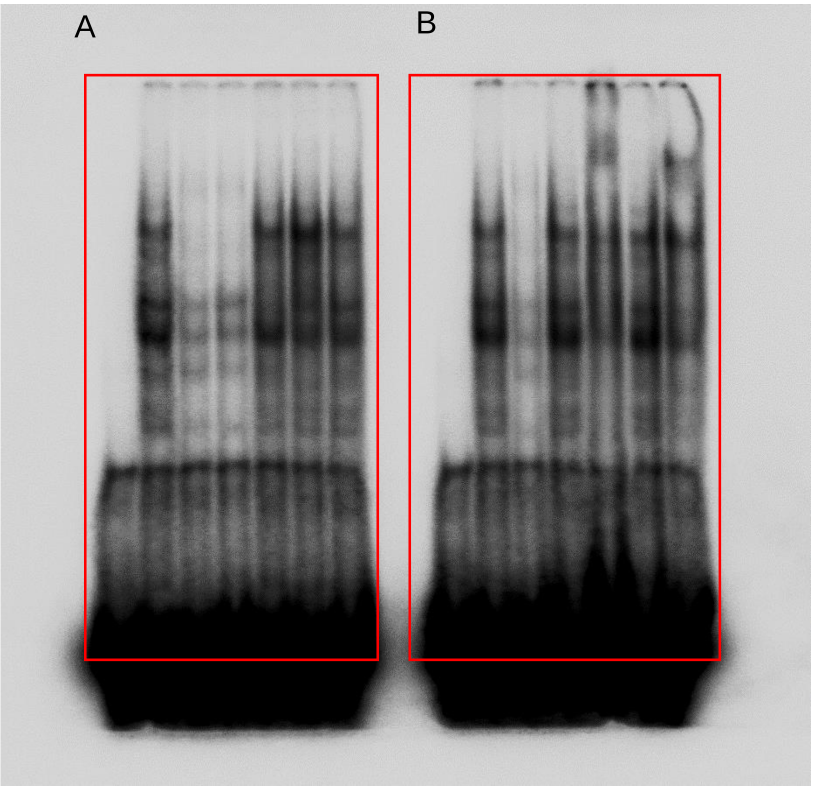

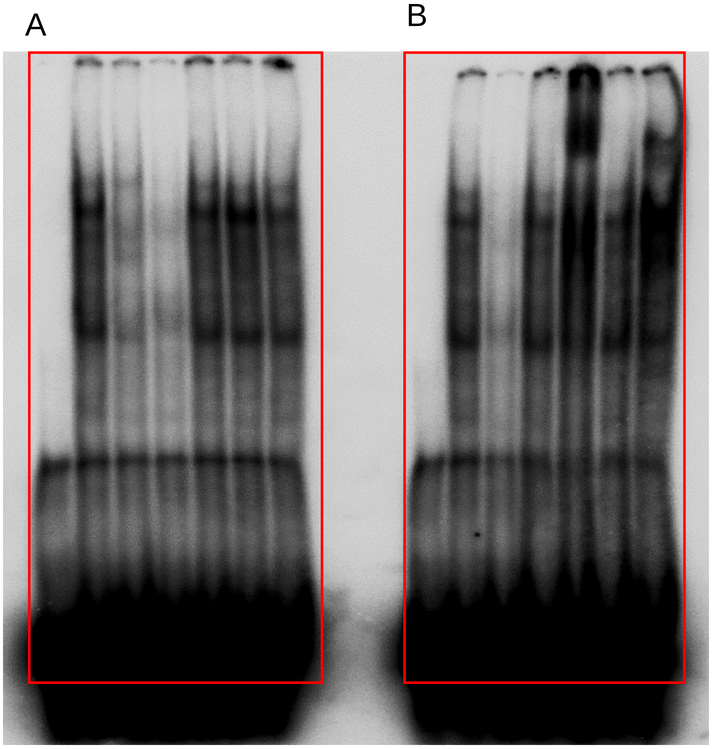

Supplementary Figure S5. Uncropped images of gels presented in the main Figure 5.

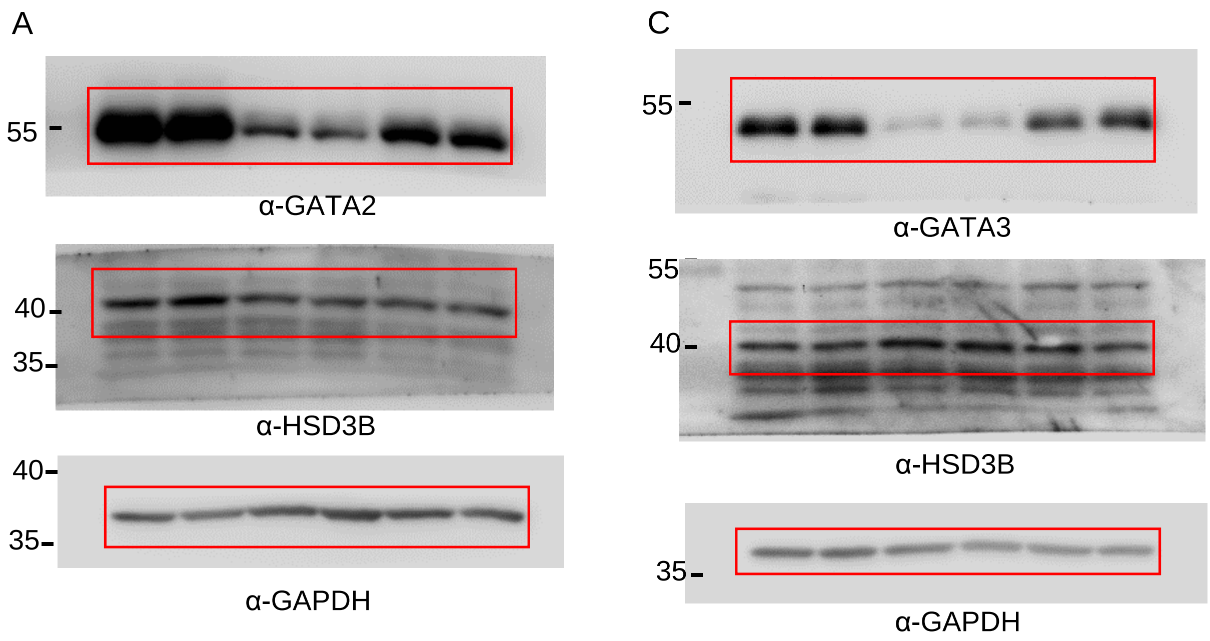

Supplementary Figure S6. Uncropped images of blots presented in the main Figure 6.
